# Case report: Long-term response to a combination of immune checkpoint inhibitors as a single treatment for multiple synchronous cancers: a case study

**DOI:** 10.3389/fimmu.2024.1487227

**Published:** 2024-12-16

**Authors:** Nour Ghammem, Herve Bischoff, Pascale Chiappa, Laura Somme, Fabien Moinard-Butot

**Affiliations:** Medical Oncology, Institut de Cancérologie Strasbourg Europe (ICANS), Strasbourg, France

**Keywords:** multiple primary malignancies, lung cancer, urothelial carcinoma, hepatocellular carcinoma, immune checkpoint inhibitor

## Abstract

**Introduction:**

Immune checkpoint inhibitors (ICIs) have revolutionized cancer therapy by enhancing the antitumor immune response. This case describes an 80-year-old male with synchronous multiple primary malignancies (MPMs), including lung metastatic hepatocellular carcinoma (HCC), and non-small cell lung carcinoma (NSCLC), and brain metastatic urothelial carcinoma, who was treated with dual ICI therapy.

**Case presentation:**

The patient, with a history of diabetes, hypertension, dyslipidaemia, well-differentiated neuroendocrine duodenal tumors and micronodular exogenous cirrhosis (Child-Pugh class A), presented with a non-invasive bladder carcinoma (pT1N0M0) resected endoscopically in December 2022. Incidentally discovered hepatic and pulmonary tumors were confirmed as primary HCC and squamous cell carcinoma of the lung (cT1bN0M0, PD-L1 expression 100%), respectively. Due to the rapid progression of pulmonary metastases secondary to HCC, dual ICI therapy (durvalumab and tremelimumab) was initiated, resulting in a partial response (>30%) according to RECISTv1.1 criteria in pulmonary and hepatic lesions. After one year of ICI therapy, cerebellar syndrome due to secondary brain lesions emerged, which was confirmed as urothelial metastases. Surgical resection of the symptomatic cerebral metastases was completed with cerebral radiotherapy, and ICIs were continued. The patient is still receiving dual ICIs.

**Discussion:**

This case highlights the crucial role of ICIs in treating MPMs. The patient’s favourable response suggests the importance of PD-L1 expression as a predictive biomarker.

**Conclusion:**

This rare case showed dual ICI therapy efficacy across multiple malignancies. Effective multidisciplinary collaboration and biomarker evaluation are crucial for managing such complex cases.

## Introduction

Multiple primary malignancies (MPM) are defined as the occurrence of two or more cancers in the same individual. The incidence of MPM ranges between 2-17%. MPM represent a significant clinical challenge because of their complex diagnostic and therapeutic implications. These malignancies can be categorized as synchronous, diagnosed simultaneously or within six months, or metachronous, diagnosed at intervals greater than six months. Risk factors for MPM include genetic predispositions, environmental exposures, previous cancer treatments, and lifestyle factors such as smoking and alcohol consumption. The increasing incidence of MPM can also be attributed to improved diagnostics and more prolonged survival of cancer patients, underscoring the need for vigilant long-term surveillance and comprehensive management strategies (1).

Over the past decade, immune checkpoint inhibitors (ICI) have revolutionized therapeutic strategies for many solid tumors by enhancing the antitumor immune response (2). These Food and Drug Administration and European Medicines Agency approved monoclonal antibodies target co-inhibitory molecules, thereby blocking the negative regulatory pathways of the immune system. ICI, widely employed in the treatment of various cancers, offer promising therapeutic options for MPM.

In the therapeutic management of metastatic hepatocellular carcinoma (HCC), ICI have demonstrated efficacy in combination with anti-angiogenic agents. The IMBrave150 trial revealed improved overall survival (OS) with the combination of atezolizumab (ICI anti-PDL1) and bevacizumab (antibody against VEGF) *versus* sorafenib (multi-kinase inhibitor) (HR 0.66; 95% CI 0.52–0.85; p <0.001) (3). The HIMALAYA trial reported that the combination of durvalumab (ICI anti-PDL1) and tremelimumab (ICI anti-CTLA4) also improved OS, with a median of 16.6 months, compared with 13.8 months for sorafenib in the first-line setting (4).

In the treatment of metastatic non-small cell lung carcinoma (NSCLC), immunotherapy alone or in combination with chemotherapy has shown significant efficacy. Compared with chemotherapy alone, combinations of chemotherapies, such as pembrolizumab (ICI anti-PD1) with platinum-based chemotherapy, improved survival outcomes (5, 6). In patients with a PD-L1 rate greater than 50%, pembrolizumab monotherapy has proven effective, as shown in the Keynote-042 study (7). In addition, the CheckMate-227 trial demonstrated superior OS with nivolumab (ICI anti-PD1) plus ipilimumab (ICI anti-CTLA4) compared with chemotherapy in the first-line treatment of metastatic NSCLC patients with PD-L1 expression above 1% (HR = 0.76; 95% CI 0.65–0.90) (8). The MYSTIC trial evaluated the combination of durvalumab (anti-PD-L1) and tremelimumab (anti-CTLA4) in metastatic NSCLC. The trial demonstrated a significant improvement in progression-free survival (PFS) with the combination therapy compared to chemotherapy, particularly in patients with high PD-L1 expression. However, the OS benefit was not consistently demonstrated in the initial analysis, with a HR for OS of 0.85 (95% CI 0.61–1.17) in the durvalumab plus tremelimumab arm versus chemotherapy (9).

Significant advancements have also been made in the treatment of metastatic bladder cancer with the introduction of ICIs. The phase III Keynote-045 trial demonstrated the efficacy of pembrolizumab monotherapy as a second-line treatment (10). Avelumab (ICI anti-PDL1) showed a survival benefit as maintenance therapy following platinum-based chemotherapy (11). The EV-302 study, which evaluated pembrolizumab in combination with enfortumab-vedotin (antibody-drug conjugate against Nectin 4) *versus* chemotherapy, reported a doubling of OS in patients receiving metastatic first-line treatment (12).

In this report, we describe a patient with multiple synchronous malignancies, including metastatic HCC, NSCLC, and urothelial carcinoma, whose treatment with an ICI doublet regimen proved effective.

## Case presentation

**Figure 1:**
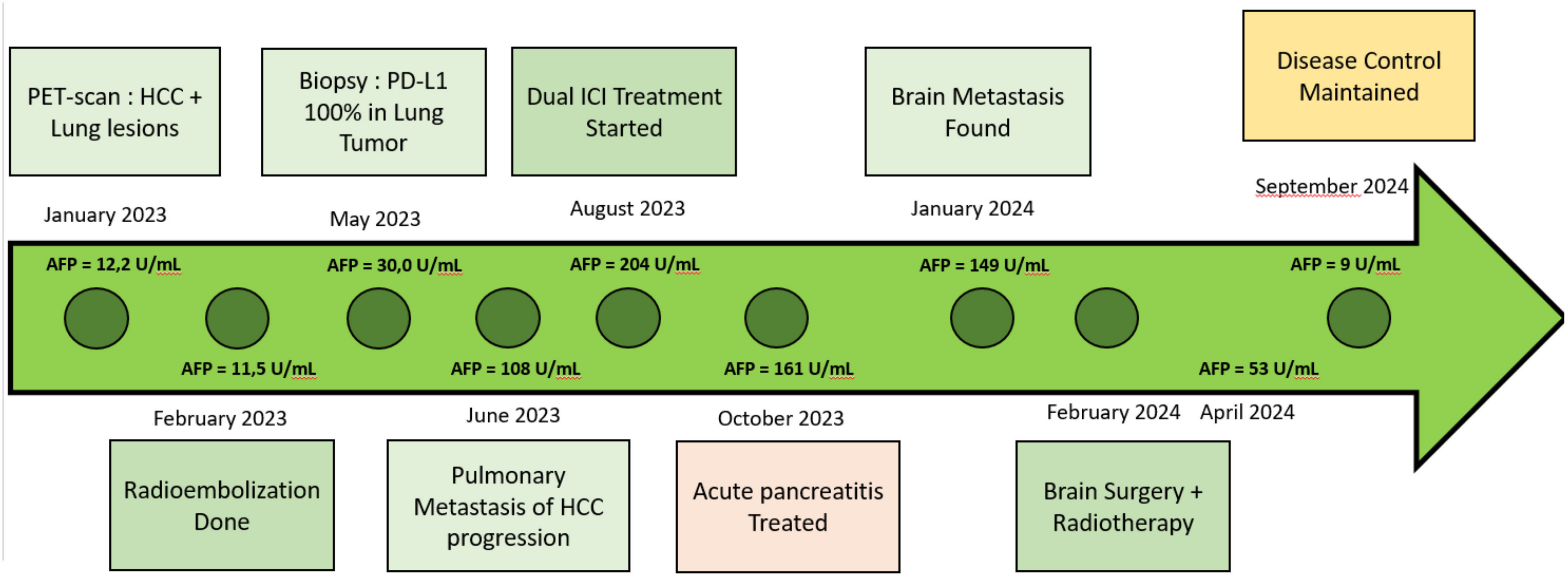
Timeline AFP, Alpha-fetoprotein; PDL1, programmed death ligand 1; HCC, Hepacellular carcinoma.

An 80-year-old male with a medical history of diabetes, hypertension, dyslipidaemia, multiple neuroendocrine duodenal tumors, and micronodular exogenous cirrhosis (classified as Child-Pugh class A) was diagnosed in December 2022 with non-invasive bladder carcinoma, classified as pT1N0M0. The patient did not receive further treatment for the bladder carcinoma. During an abdominal ultrasound performed for cirrhosis monitoring, a bilocular hepatic tumor with an encapsulated appearance was discovered in segment VI of the right liver, along with an endoportal tumor thrombosis. Subsequent ^18^F-FDG PET-Scan on January 2023 confirmed hypermetabolic tissue lesions suggestive of primary HCC and a suspicious spiculated pseudo-nodular lesion in the right lung. Alpha-fetoprotein (AFP) levels were elevated to 12.217 U/ml, surpassing the normal range (<10 U/ml) (**Table 1**). Following hepatic radioembolization in February 2023, a liver MRI revealed a mixed response with a partial response in the right liver, indicating a tumor response in the endovenous system and progression of the disease due to regional invasion of the biliary ducts (**Figure 1**).

**Table 1:** key laboratory test results, monitoring during treatment.

Date	AFP (U/ml)	ALAT (U/L)	ASAT (U/L)	Bilirubine (µmol/L)	Platelet count (x10^3^/µL)	Creatinine (µmol/L)
Jan 2023	12.217	22	24	11.9	178	120
Feb 2023	11.475	23	28	18.2	162	116
May 2023	30	27	34	8.9	151	133
June 2023	108	29	22	10.5	149	111
Aug 2023	204	29	21	9.1	164	102
Oct 2023	161	26	19	6.9	222	131
Jan 2024	149	25	20	10.6	153	110
Feb 2024	53	30	17	12.5	145	102
Sept 2024	9	29	16	8.9	163	106

AFP, Alpha-fetoprotein; ALT, Alanine aminotransferase; AST, Aspartate aminotransferase.

**Figure 2:**
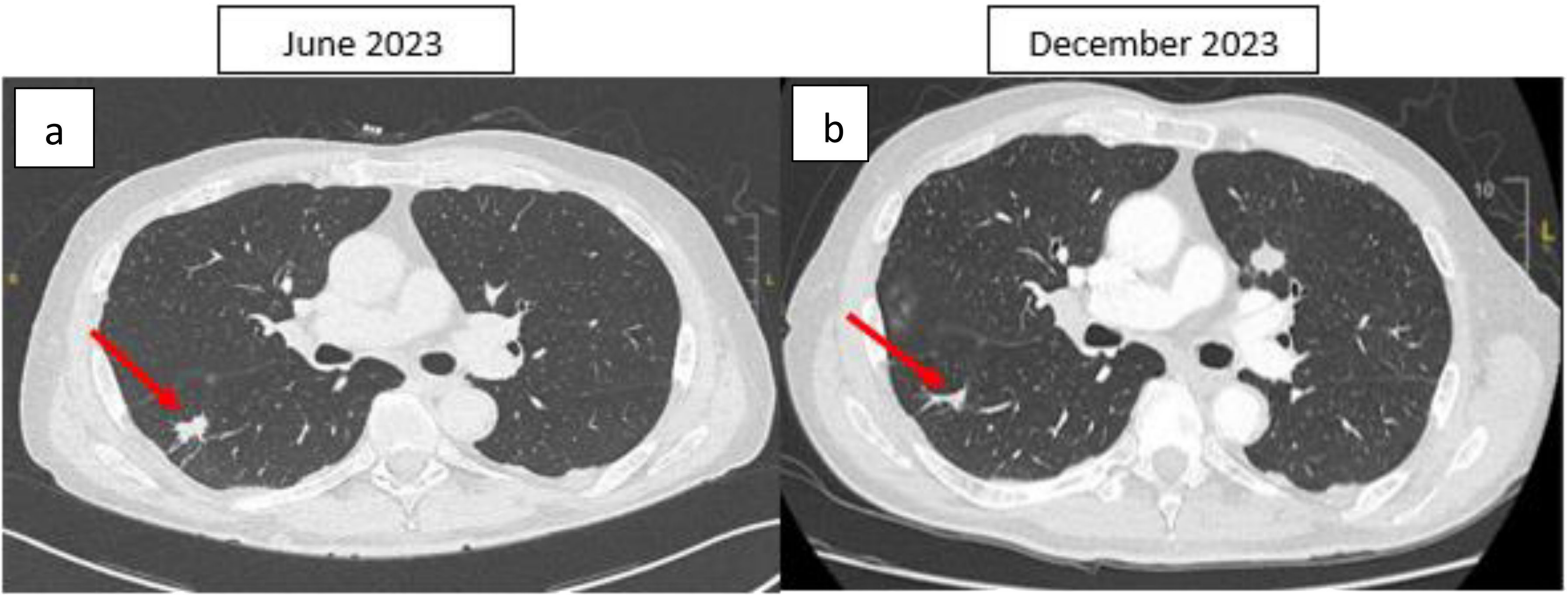
Thoracic CT scan, axial section: **(A)** Spiculated nodule in the apical segment of the right lower lobe initially measuring 20 × 11 mm, with some spicules reaching the fissural and parietal pleura. **(B)** After four months of ICI therapy, the nodule was reduced to 15 × 3 mm. The right lung lesion demonstrates a reduction in size between the June 2023 and December 2023 CT scans. However, a growing nodule is observed in the left lung on the December 2023 scan. This may be explained by the fact that the June 2023 scan was performed prior to the initiation of immunotherapy (which began in August 2023). As a result, the left lung nodule (HCC metastases) could have continued to progress during the interval between the scans, before the therapeutic effects of immunotherapy were fully realized. This temporal difference in treatment response may account for the observed discrepancies in lesion size. The red arrow indicates the lung tumor.

**Figure 3:**
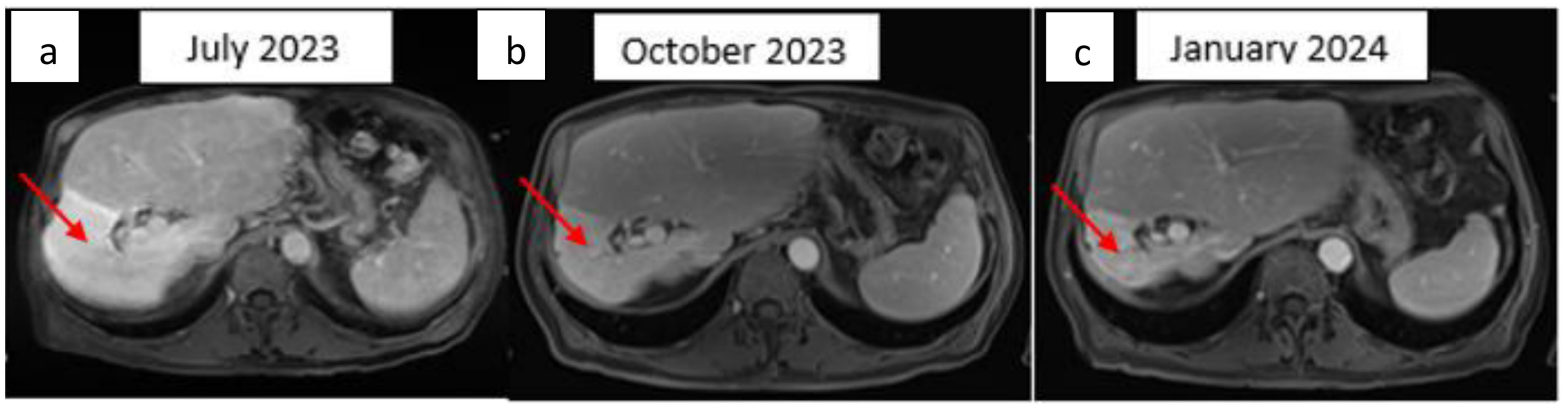
Hepatic T1 MRI image after gadolinium injection for three min. Axial image: **(A)** Bilocular hepatic tumor with an encapsulated appearance in segment VI of the right liver, initially measuring 69 × 68 × 81 mm. **(B)** Partial response with a 25% reduction after three months of ICI therapy. **(C)** Partial response with 50% reduction mm after six months of ICI therapy. The red arrow indicates the hepatic tumor.

In May 2023, a CT-guided biopsy of the suspicious pulmonary lesion identified a squamous cell lung carcinoma in the right lower lobe, classified as cT1bN0M0 (**Figure 2A**), with a 100% PD-L1 expression level. The multidisciplinary team meeting recommended stereotactic radiotherapy. A thoracic CT scan in June 2023, performed prior to radiotherapy, revealed the appearance of pulmonary nodules. A lung biopsy confirmed pulmonary metastatic involvement of the HCC. The patient did not undergo stereotactic radiotherapy for his NSCLC after confirmation of metastasis. Owing to the rapid progression of pulmonary tumors secondary to hepatic carcinoma, first-line treatment with dual ICI, consisting of durvalumab and tremelimumab, was started in August 2023 (**Figure 1**). This treatment led to a partial pulmonary (**Figure 2B**), hepatic response (**Figure 3**), and decreased AFP level to 9 U/ml (**Table 1**).

After the patient had received four cycles of ICI with durvalumab and tremelimumab, he developed CTCAE grade I acute pancreatitis. This complication was detected based on lipase levels, which were significantly higher than the normal range. Upon diagnosis, the patient was promptly treated with corticosteroids at a dose of 1 mg/kg/day, which led to a marked improvement in his clinical condition. The patient had a complete recovery from the pancreatitis, with normalization of lipase levels within two weeks of initiating steroid therapy. Regarding immune-related adverse events (irAEs), the patient did not experience any other immune-related toxicities.

**Figure 4:**
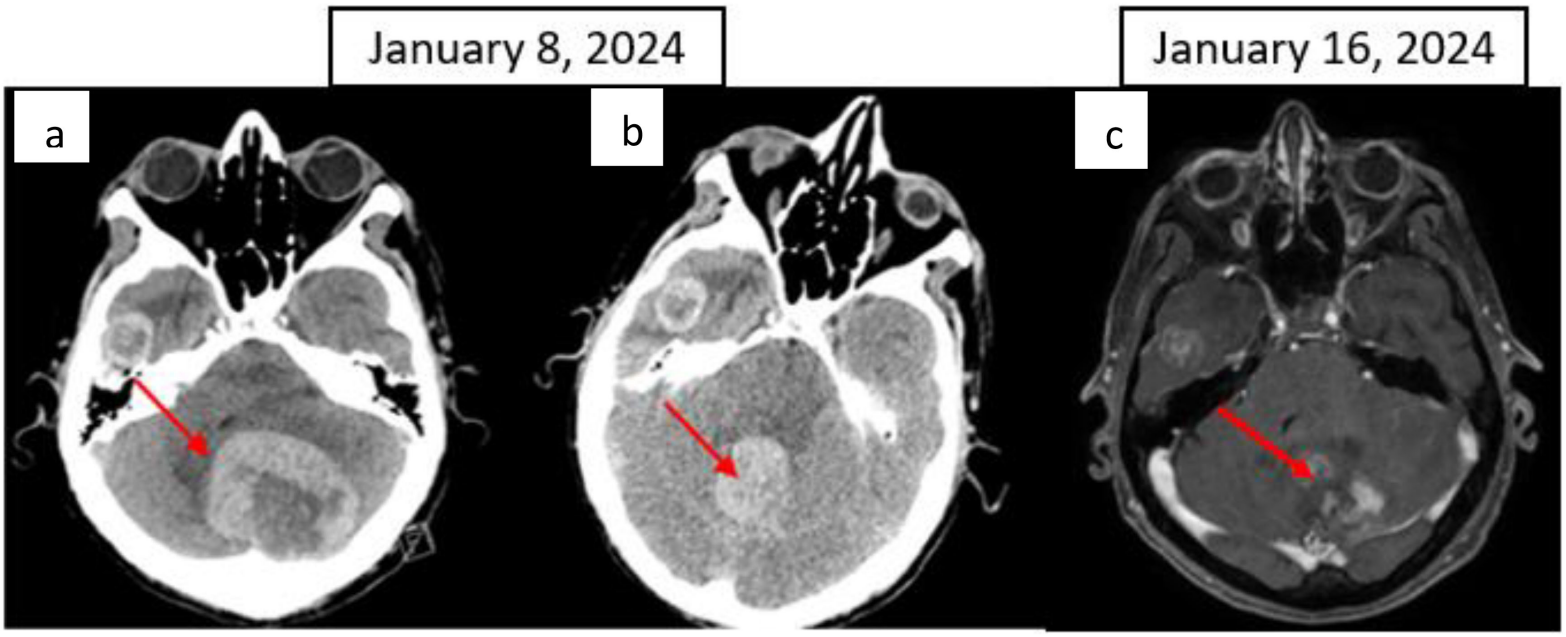
CT scan: **(A)** Left cerebellar metastasis measuring 55 × 51 mm; **(B)** right temporal metastasis with a necrotic centre measuring 24 × 18 mm; **(C)** Brain T1 MRI image after gadolinium injection, axial image. The red arrow indicates the cerebellar metastasis.

After six months of ICI therapy, the patient developed cerebellar syndrome, and further imaging revealed secondary brain lesions: a 17 mm lesion in the right temporal lobe and a 49 mm lesion in the left cerebellum (**Figure 4**). Following surgical resection of the cerebellar tumor, histological analysis revealed carcinoma proliferation with GATA3 positivity, consistent with the patient’s primary urothelial carcinoma. Immunostaining revealed a combined positive score (CPS) of 40. Post-surgery, the patient received cerebral radiotherapy to the operative bed in the left cerebellum and the right temporal lesion under stereotactic conditions. It was decided to continue with ICI, which have permitted disease control for 12 months. The patient is still receiving ICI therapy (**Figure 1**).

## Discussion

This rare case reported an extended response to dual ICI therapy in a patient with synchronous pulmonary metastatic hepatocarcinoma, squamous lung cancer and brain metastatic urothelial carcinoma. Focal treatment for brain progression allowed the continuation of ICI therapy.

Managing MPM poses a significant clinical challenge because of their diverse tumor characteristics and treatment responses. There are currently no standard guidelines for the therapeutic management of MPM. These rare occurrences underscore the complexity in designing effective therapeutic strategies that address each cancer type without compromising patient outcomes (1). Our patient’s case involving synchronous hepatocellular carcinoma, non-small cell lung cancer, and urothelial carcinoma highlights the intricate nature of MPM and the necessity for tailored treatment approaches (13). ICI could revolutionize MPM treatment by harnessing the immune system against tumors (14). As reported in this case, treatment with durvalumab and tremelimumab demonstrated extended efficacy across multiple cancers, emphasizing the evolving role of ICI in managing complex oncological scenarios (15).

The management of MPM in an elderly patient, particularly one with significant comorbidities such as diabetes, hypertension requires a careful balance between efficacy and safety. In this case, the patient’s relatively well-preserved liver function (Child-Pugh A) and good overall performance status were key factors that allowed the administration of dual ICI, despite his advanced age. While ICI, such as durvalumab and tremelimumab, have demonstrated substantial clinical efficacy in several cancers, their use in elderly patients, particularly those with liver cirrhosis and other comorbidities, remains complex (16, 17). The absence of significant immune-related toxicities other than mild pancreatitis, which was successfully managed, underscores the feasibility of ICI therapy in this population. This favorable outcome encourages further exploration of dual ICI therapy in elderly MPM patients, as it may offer prolonged disease control, even in complex cases (18). However, it also highlights the need for close monitoring of irAEs, especially given the increased vulnerability of older adults to treatment-related toxicities (19). The patient’s favorable tolerance and response to dual therapy suggest that age and comorbidities should not be definitive contraindications to aggressive immunotherapy in MPMs. Instead, these factors should be carefully evaluated when making individualized treatment decisions (20).

While single-agent immunotherapy has proven effective in certain cancers, its use is often limited by factors such as suboptimal response rates and the development of resistance, particularly in tumors with lower mutational burden or complex immune escape mechanisms (21). For instance, monotherapy with PD-1/PD-L1 inhibitors is associated with variable responses in cancers like NSCLC and HCC (22). In contrast, combination immunotherapy has emerged as a promising strategy to overcome these limitations by targeting multiple immune checkpoints, thereby enhancing the durable immune response (23). The use of dual ICI regimens, such as durvalumab and tremelimumab, has shown improved efficacy in treating complex cases, as evidenced by our patient with MPM (24). However, while combination therapies offer increased treatment efficacy, they also come with an elevated risk of irAEs. Thus, careful patient selection and monitoring are crucial to maximize the benefits of combination immunotherapy while minimizing potential toxicities (25).

Predictive biomarkers such as PD-L1 expression and tumor mutational burden (TMB) are crucial in guiding ICI therapy decisions and predicting treatment responses across different treatment responses cancer types (26). In our study, high PD-L1 expression in lung cancer cells correlated with favourable responses to durvalumab and tremelimumab, highlighting PD-L1 as a potential biomarker for ICI efficacy prediction. Similar findings in NSCLC studies support the notion of enhanced ICI response in tumors with elevated PD-L1 expression (27, 28). However, while PD-L1 expression has shown predictive value in some cancers, its role as a reliable biomarker for combination immunotherapies, especially in HCC, remains less clear, and the evidence for its utility in guiding dual ICI treatment is still evolving. For MPM, the immune environment, with markers of response to immunotherapy, such as the CPS, has a greater impact than the tumor histological type on the introduction of 1^st^-line immunotherapy. Despite an initial diagnosis of non-invasive bladder cancer, our patient developed brain metastases, illustrating the complexities of managing metastatic patterns and the varying responses of metastatic sites to ICI therapy (29). Reports in the literature indicate mixed outcomes in controlling intracranial disease with ICIs, indicating both potential and limitations in treating brain metastases (30). Recent studies suggest that although ICIs can be effective in controlling extracerebral disease, the response to brain metastases may be less predictable, necessitating the combination of focal therapies such as radiotherapy to manage intracranial progression while continuing ICI treatment for systemic disease (31). Brain focal therapies remain a complementary therapeutic option to continue ICIs, especially in cases of extracerebral disease control with exclusive brain progression (32). The evolution from non-invasive bladder cancer to muscle-invasive evolution with exclusive brain metastases underscores the unpredictable nature of cancer progression and the need for close monitoring and adaptive treatment strategies tailored to the individual patient’s disease trajectory.

## Conclusion

Integrating ICI into MPM management represents a transformative approach in oncology, emphasizing personalized treatment strategies and the pivotal role of immunological biomarkers in optimizing therapeutic outcomes. Further research is warranted to elucidate the underlying mechanisms governing differential ICI responses across diverse cancer types and metastatic sites, paving the way for more effective and tailored therapies for patients with complex malignancies.

## Data Availability

The original contributions presented in the study are included in the article/supplementary material. Further inquiries can be directed to the corresponding author.
